# Addressing Health Disparities in ADRC Brain Donation: A Community‐Driven Initiative in Upper Manhattan

**DOI:** 10.1002/alz70860_106276

**Published:** 2025-12-23

**Authors:** Gelanys Castro, Lawrence S. Honig, Howard F Andrews, David P Merle, Naara Ramirez‐Estevez, Violeta Epstein, Sigrid Gabler, Miguel Arce Renteria, Tarialy Hernandez, Donovan Laing, Andrew F. Teich, James M. Noble

**Affiliations:** ^1^ Columbia University, New York, NY, USA; ^2^ Columbia University Irving Medical Center, New York, NY, USA; ^3^ Taub Institute for Research on Alzheimer's Disease and the Aging Brain, Columbia University, New York, NY, USA; ^4^ Mailman School of Public Health, Columbia University, New York, NY, USA; ^5^ Department of Neurology, Columbia University Medical Center, New York City, NY, USA; ^6^ Taub Institute for Research on Alzheimer's Disease and the Aging Brain, New York, NY, USA; ^7^ Department of Pathology and Cell Biology, New York, NY, USA; ^8^ Taub Institute for Research on Alzheimer's Disease and the Aging Brain, Columbia University, New York, NY, USA

## Abstract

**Background:**

In the United States, under‐representation of Hispanic and Black participants in medical research is an ongoing concern, particularly in Alzheimer's disease (AD) and related dementias (ADRD). Knowledge gaps follow with respect to AD/ADRD epidemiology, pathobiology, and treatments, and similar gaps are known in post‐mortem studies. The Alzheimer's Disease Research Center (ADRC) at Columbia University Irving Medical Center (CUIMC) aims to address AD/ADRD health disparities by facilitating engagement and enrollment of participants at our center including the local Washington Heights community. Here we report on efforts to improve brain donation discussions and commitments as part of ADRC participation, and the relationship with completed brain donations.

**Method:**

In the Columbia ADRC, authentic community engagement is prioritized including Spanish and English bilingualism in all circulated materials, research assistants, its brain donation coordinator, and several clinicians including the clinical and outreach core leaders. Brain autopsy is not an inclusion criteria for ADRC enrollment, but is a planned point of discussion with each participant during visits. A process to track participant initial interest in brain donation began in 2000 and prompts follow‐up calls with brain donation coordinators.

**Results:**

Since 2005, the Columbia ADRC clinical core has facilitated 1,663 brain donations into the New York Brain Bank at CUIMC across multiple studies focused on aging and neurodegeneration. In the ADRC, 405 (49.6% of 814 known decedents) have been autopsied. Of 2356 UDS‐evaluated persons, 1127 (47.8%) had a documented brain donation discussion initiated with a clinician, leading to 634 follow‐up discussions (56.3% of 1127) and 445 agreements (70.2% of 634). Among 297 UDS‐characterized decedents with a documented brain donation conversation, 215 (72.4%) later donated; documented intent was associated with 201 donations (81.0% donation rate). Among those with documented brain donation conversations, 73.3% of Whites, 60.0% of Hispanics, and 55.1% of Blacks have expressed intent to donate.

**Conclusions:**

Initiating and tracking brain donation conversations is associated with an increased likelihood to donate. The majority of each race‐ethnic group at our center expressed intent to donate following brain donation discussion. Initiatives informed by community based participatory research demonstrate feasible approaches to improving brain donation rates among underrepresented populations.

